# Metabolomics in the Context of Plant Natural Products Research: From Sample Preparation to Metabolite Analysis

**DOI:** 10.3390/metabo10010037

**Published:** 2020-01-15

**Authors:** Mohamed A. Salem, Leonardo Perez de Souza, Ahmed Serag, Alisdair R. Fernie, Mohamed A. Farag, Shahira M. Ezzat, Saleh Alseekh

**Affiliations:** 1 Department of Pharmacognosy, Faculty of Pharmacy, Menoufia University, Gamal Abd El Nasr st., Shibin Elkom, Menoufia 32511, Egypt; 2 Max Planck Institute of Molecular Plant Physiology, Am Mühlenberg 1, 14476 Potsdam-Golm, Germany; LPerez@mpimp-golm.mpg.de (L.P.d.S.); Fernie@mpimp-golm.mpg.de (A.R.F.); 3 Pharmaceutical Analytical Chemistry Department, Faculty of Pharmacy, Al-Azhar University, Cairo 11751, Egypt; Ahmedserag777@azhar.edu.eg; 4 Center of Plant Systems Biology and Biotechnology (CPSBB), Plovdiv 4000, Bulgaria; 5 Pharmacognosy Department, Faculty of Pharmacy, Cairo University, Cairo 11562, Egypt; Mohamed.farag@pharma.cu.edu.eg (M.A.F.); shahira.ezzat@pharma.cu.edu.eg (S.M.E.); 6 Chemistry Department, School of Sciences & Engineering, The American University in Cairo, New Cairo 11835, Egypt; 7 Department of Pharmacognosy, Faculty of Pharmacy, October University for Modern Sciences and Arts (MSA), Giza 11787, Egypt

**Keywords:** metabolomics, plant natural products, drug discovery, metabolite extraction, liquid chromatography, gas chromatography, mass spectrometry, NMR

## Abstract

Plant-derived natural products have long been considered a valuable source of lead compounds for drug development. Natural extracts are usually composed of hundreds to thousands of metabolites, whereby the bioactivity of natural extracts can be represented by synergism between several metabolites. However, isolating every single compound from a natural extract is not always possible due to the complex chemistry and presence of most secondary metabolites at very low levels. Metabolomics has emerged in recent years as an indispensable tool for the analysis of thousands of metabolites from crude natural extracts, leading to a paradigm shift in natural products drug research. Analytical methods such as mass spectrometry (MS) and nuclear magnetic resonance (NMR) are used to comprehensively annotate the constituents of plant natural products for screening, drug discovery as well as for quality control purposes such as those required for phytomedicine. In this review, the current advancements in plant sample preparation, sample measurements, and data analysis are presented alongside a few case studies of the successful applications of these processes in plant natural product drug discovery.

## 1. Introduction

Nature provides a rich source of numerous bioactive compounds that have been extensively employed in traditional medicine since time immemorial [1]. In recent years, the Food and Drug Administration (FDA) has approved an impressive number of modern drugs that are also natural products or directly derived therefrom [2]. These mostly constitute compounds belonging to secondary metabolic pathways with prominent examples including taxol from *Taxus brevifolia*, vinblastine from *Catharanthus roseus*, doxorubicin from *Streptomyces peucetius,* and cyclosporine from *Tolypocladium inflatum* [3,4]. The first step in the discovery of lead compounds from natural sources is the release of bioactive metabolites from their biomass through various extraction techniques viz., supercritical fluid extraction [5,6], microwave-assisted and ultrasonic-assisted extraction [7], molecular distillation methods [8], and membrane separation technology [9]. Moreover, bioassay-guided fractionation using chromatographic methods such as preparative high performance liquid chromatography (HPLC) is applied for the isolation and purification of active metabolites from their crude extracts [10]. Further technologies such as nuclear magnetic resonance spectroscopy (NMR), mass spectrometry (MS), and ultraviolet-visible spectroscopy (UV–Vis) have allowed the detailed characterization and ultimately, the structural elucidation of these agents [11]. Finally, the bioactivity (effects in cell lines, animal models, and human volunteers) is investigated for assessing the pharmacological potential of the candidate compounds. Nevertheless, some pitfalls were observed when employing this classical approach for lead compound discovery, where degradation or chemical modification of the bioactive compounds during the process of isolation and purification often occurs. Furthermore, important biological information that was present in the original extract might be lost during activity-guided fractionation as the samples are not fully analyzed [12]. Moreover, better therapeutic effects were reported when using the whole extracts, as practiced in traditional medicine, rather than a single-compound based remedy. This effect could be attributed to the synergy between bioactive components (e.g., studies showed the synergistic effects of different plant extracts and doxorubicin in cancer treatment [13], Apocynaceae plants and antibiotics against *Acinetobacter baumannii* [14], and catechin and resveratrol as antioxidants [15]).

Unlike the classical approach in natural products research, metabolomics experiments offer an improved expedited route for drug discovery [16]. The basic goal of metabolomics is to provide a comprehensive qualitative and/or quantitative analysis of all metabolites present in a living system [17]. Interestingly, this concept could be extended in natural product drug discovery via studying the relationship between the whole metabolome of natural-derived remedies and their biological effects [18]. Implementing such an approach not only overcomes the aforementioned pitfalls of the classical techniques used in natural product research, but also provides a broader insight of the biochemical status and gene functions of the studied organisms. Furthermore, the signature between specific compounds in the metabolome and their bioactivity could also be revealed, aided by advanced bioinformatics tools, a process that can be highly useful in pharmacological standardization and biological fingerprinting of natural extracts [19]. The workflow of metabolomics experiments involves an efficient extraction of these endogenous metabolites to be subjected for qualitative and quantitative analysis. However, unlike other omics technologies, no single analytical platform is capable of analyzing all metabolites simultaneously due to their extreme complexity and huge chemical diversity. Recent developments in analytical chemistry platforms such as hyphenating mass spectrometry with gas chromatography (GC), liquid chromatography (LC) or capillary electrophoresis (CE), and nuclear magnetic resonance (NMR) spectroscopy have led to a highly efficient set up for metabolome analysis [20], however, these do not yet reach comprehensibility [21]. Although huge datasets are generated from these instruments, the evolution of chemometrics and multivariate data analysis algorithms provides a powerful tool for extracting useful information from such high dimensionality results [22]. They enable, for example, the detection of compounds that correlate to the medicinal efficacy in test animal or human systems based on their analytical spectral fingerprints [23]. Moreover, pattern recognition and classification algorithms have also allowed the implementation of metabolomics as an effective tool for the quality control of herbal medicinal products [24,25]. Nevertheless, metabolite identification remains the most challenging aspect of metabolomics experiments [26]. Large mass spectral and NMR spectral databases have been created to untangle such problems [27]. Furthermore, bioinformatics tools based on molecular networking such as GNPS [28] and MetGem [29] have been implemented not only to assign known metabolites from their complex mixtures, but also to elucidate the chemical structures of novel compounds of interest.

This review will discuss the recent developments of metabolomics in the context of plant natural product drug discovery including current advances in sample preparation techniques and analytical profiling platforms. In addition, computational tools employed for metabolomics data processing and metabolites identification are reviewed. Finally, some successful applications of metabolomics in the identification of bioactive agents and in the quality control of natural products are outlined, and an outlook for the increasing use of metabolomics in these fields is provided. 

## 2. Sample Preparation for Metabolomic Studies

**Figure 1:**
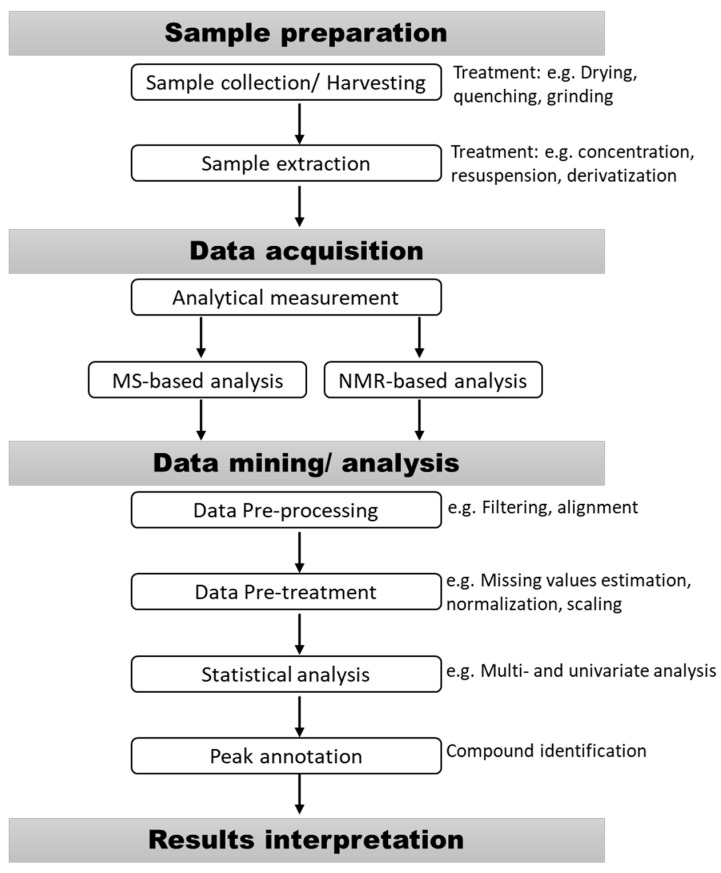
Key steps of a plant metabolomics study. Additional steps can be performed or modified in certain approaches based on the analytical methods or point of interest.

In metabolomics studies, biological samples are collected, extracted, measured, and finally, the resulting data are analyzed (Figure 1). Sample preparation is a crucial step in metabolomics as it greatly affects the reliability of the metabolomics results. Minor changes in the sample collection, extraction, or storage greatly affect metabolite stability and hence can lead to major changes in the observed metabolome. Metabolomics samples have to be collected uniformly and rapidly to avoid changes due to the fast enzymatic turnover rate [30]. The ultimate aim is to minimize the biologically-irrelevant changes resulting from sample processing. Improper handling of biological samples is the most likely source of bias in metabolomic studies [30]. In order to validate plant metabolomics studies, the minimum parameters related to experimental design, sample extraction to data analysis should ideally follow the Metabolomics Standards Initiative (MSI) [31].

### 2.1. Sample Collection

A wide range of biological samples have been extensively reported in metabolomics studies. This includes tissues (animal-and plant-derived tissues), fluids (such as urine, whole blood, serum, sweat, cerebrospinal fluid, breast milk, amniotic fluid, saliva, etc.), and cell cultures (human, animal, plant, algae, etc.) [32,33]. Such complex samples have different matrices, and therefore, require different sample preparation protocols. Here, we focus on natural products that are mainly derived from plant origin, while also giving a general overview of other tissues. Depending on the natural abundance of some metabolites and the level of detection, various amounts of biological material per sample have to be considered. Usually, 1–100 mg of tissue, 10−250 μL of fluids, or 10^5^−10^7^ cells per each biological replicate is required [33]. A minimum number of 3–5 biological replicates is recommended for each condition in metabolomic studies [31]. Biological replicates (parallel measurements of samples from different individuals) rather than technical replicates (repeated measurements of the same sample) are to be considered [34].

### 2.2. Harvesting Methods

Sample freezing methods such as using dry ice or liquid nitrogen are highly recommended during the harvesting of fresh samples to avoid enzyme-induced metabolic changes [31]. Removing unwanted components such as soil particles is also recommended before collection. Long-term storage of samples prior to extraction should, however, be avoided. For short term storage (e.g., for few days up to two weeks), samples can be kept in liquid nitrogen, dry ice, or a −80 °C freezer [31]. Prior to extraction, the harvested samples can be exposed to processing methods such as lyophilization, cell lysis, and/or grinding, depending on the biological material. The conditions related to cultivation parameters, collected tissue type, seasonality, developmental stage, harvesting time, and sample processing should be reported for each condition since metabolites are greatly affected by such parameters [35], with environmental aspects being reported to induce both qualitative and quantitative variations in the metabolite composition of both plant primary and secondary metabolites [36]. 

### 2.3. Sample Extraction

Unlike the genome and proteome, which can be captured using a single extraction protocol, the metabolome is difficult to capture within a single solvent due to the diverse chemistry of metabolites [37]. Therefore, complex biological samples, which as stated above, have different matrices, and therefore, require different extraction protocols. Generally, metabolites are preferentially extracted with the solvent following the rule of thumb “like dissolves like”. Polar and semi-polar metabolites can be extracted with hydrophilic solvents such as hydro-alcoholic solutions, while, lipids can be extracted with more hydrophobic solvents. Several protocols for metabolome extraction have been developed and extensively reviewed [32,33,35,38,39,40]. The selected method must be rapid and efficient, whilst at the same time, cover a wide range of target metabolites, thus maintaining a high level of precision and accuracy. 

Liquid–liquid fractionation provides significant simplifying steps compared to single extraction methods [41]. Partial purification of each fraction is achieved via removing interfering compounds such as hydrophobic molecules that are enriched in lipophilic solvents and more polar metabolites in hydrophilic solvents [42]. While different extraction protocols are varied with regard to the selected solvent(s) and the amount that is to be used, most protocols include a deproteinization step. The presence of proteins in the analyzed samples can severely affect the instrument’s accuracy, precision, and lifetime [43]. Extraction efficiency can usually be enhanced by selecting an extraction solvent mixture consisting of one or two steps compared to single extraction solvent [33]. Liquid–liquid extraction methods were based on the so called gold standard extraction protocols of the ‘Folch’ and ‘Bligh and Dyer’ methods utilizing chloroform/methanol mixture in different proportions [44,45]. Liquid–liquid extraction using chloroform/methanol/water have been used for the analysis of lipids and polar metabolites [46,47,48,49]. As a cleaner and safer solvent, methyl *tert*-butyl ether (MTBE) has also been used as an alternative to chloroform for liquid–liquid extraction [50], particularly for the recovery of metabolites and lipids from bacterial, plant, algal, flies, and diverse mammalian samples [41,42,51,52,53,54,55,56,57,58,59,60]. The use of isotope-labeled internal standards during sample preparation is recommended to assess matrix effects [39,61]. For the selection of suitable solvent(s) and an extraction method that is suitable for different metabolite classes, readers are referred to the available extraction protocol reviews [32,35,39,40,62,63].

### 2.4. Extract Concentration, Dilution, Enrichment, and Re-Suspension

In addition to liquid–liquid extraction, other methods such as organic solvent precipitation as well as single step or multiple step solid-phase extraction (SPE) have also been used to partially purify samples prior to analytical measurement [64]. After sample extraction, solvents are evaporated to concentrate the metabolites. Samples are typically concentrated in a vacuum concentrator at room temperature without heating. The use a nitrogen flow evaporator is recommended in cases where metabolites are sensitive to oxidative modifications as, for example, some lipids. Finally, the dry extracts are typically resuspended in an analysis-compatible solvent [43]. Dried samples can be subjected to further steps such as derivatization before GC/MS analysis [49]. Short term storage of extracted liquid samples, even at low temperature (−20 °C), is not recommended, however, samples can be stored, if necessary, in a dry state for a short time prior to analysis [65].

## 3. Analytical Methods for Metabolites Analysis

The diversity of metabolites in the plant kingdom is staggering; a commonly quoted estimate is that plants produce somewhere between 100,000 and one million metabolites [66,67]. Metabolite measurements have been carried out for decades because of the fundamental regulatory importance of metabolites as components of biochemical pathways, the importance of certain metabolites in the human diet, and their use as diagnostic markers for a wide range of biological conditions including disease and response to chemical treatment [37]. There are wide ranges of metabolomics approaches, which ultimately aim to measure the entire small molecule complement of the cell. Current metabolomics strategies are mainly reliant on four major approaches: gas chromatography-mass spectrometry (GC-MS), liquid chromatography–mass spectrometry (LC-MS), capillary electrophoresis–mass spectrometry (CE-MS), and nuclear magnetic resonance (NMR) spectroscopy. A number of detailed protocols [49,63,68,69], in addition to several excellent technical reviews [70,71] regarding the utilization of these analytical tools in metabolomics experiments, have been published, however, a brief technical overview of these four major methods is provided herein.

### 3.1. Gas Chromatography-Mass Spectrometry (GC-MS)

GC-MS has been one of the most popular metabolomics techniques to measure the levels of volatile and semi-volatile organic compounds in a wide variety of samples. In GC-MS, polar metabolites are derivitized to render them volatile and then separated by GC. Various derivatization methods such as alkylation, acylation, methoximation, trimethylsilylation, and silylation can be used [72,73]. Electron impact ionization results in highly reproducible fragmentation patterns that are essential for large-scale experiments [37]. A great advantage of GC-MS is that it is both relatively sensitive and highly robust, and can routinely and reproducibly measure hundreds of analytes across thousands of samples [72,74,75,76]. That said, a technical challenge in GC-MS profiling is to separate each metabolite signal from overlapping peaks in the raw GC-MS chromatogram [77]. Several peak-picking software packages equipped with sophisticated peak deconvolution functions are available to deal with a relatively high-throughput data of thousands of samples [77]. In addition, various databases have been developed to aid in assigning structures to spectral peaks observed in metabolomics experiments [78,79,80]. Furthermore, limitations of GC-MS have recently been improved by the development of two-dimensional GC × GC coupled with high resolution mass spectrometry [81,82,83,84]. In this approach, two columns with different properties (non-polar vs. polar) are connected through a modulator, allowing further separation of compounds that co-elute from the first column, thereby giving rise to enhanced resolution and peak capacity [85,86]. The GC-MS method has had myriad applications in plant, pharmacological, and medical metabolomics studies, and is considered as one of most suitable techniques of the accurate determination of primary metabolites, however, it is severely compromised in measuring some secondary metabolites. Several classes of volatile and non-volatile metabolites such as phenolics, alkaloids, and terpenoids have been analyzed and identified by GC-MS [79,87].

### 3.2. Liquid Chromatography-Mass Spectrometry (LC-MS)

Liquid chromatography-mass spectrometry (LC-MS) has become the most comprehensive technique to measure a wide range of diverse metabolites. Unlike GC-MS, it does not require prior sample treatment, and crude extracts obtained by simple extraction can be introduced directly to the LC-MS. The choice of columns including reversed phase, ion exchange, and hydrophobic interaction provides metabolite separation on the basis of differential chemical properties. Nowadays, reversed-phase columns such as C18 or C8 are the most commonly used for LC gradient separation [63]. The development of ultra-performance LC rendered the technique even more powerful with regard to resolution, sensitivity, and throughput. LC-MS is a unique method for measuring plant secondary metabolites such as flavonoids and alkaloids [88,89,90], membrane lipids (lipidomics) [60,91,92], and primary metabolites such as amino acids [93]. Despite the fact that LC-MS is the most comprehensive technique in hand, LC-MS by no means approaches the metabolic complement of a typical plant cell [21]. This is mainly due to the poor availability of standard compounds for secondary metabolites [94]. In the past years, a considerable improvement in the number of metabolites that can be measured and annotated has been realized, which is mainly due to the improved machine performance afforded by the development of Ultra-Performance Liquid Chromatography (UPLC) coupled with high-resolution mass analysis methods such as time-of-flight (TOF) MS, Fourier transform (FT) MS, and Orbitrap-based MS [88,90,95,96]. In addition, this improvement has relied on increased efforts in the collection of standard compounds and sharing of reference extracts [97,98,99] for use in peak annotation authentication and by the increased sophistication of computational approaches for compound annotation (see the web resources listed in [27,100]). 

### 3.3. Capillary Electrophoresis-Mass Spectrometry (CE-MS)

Capillary electrophoresis (CE) separates polar and charged compounds on the basis of their charge-to-mass ratio [74]. CE offers fast and high-resolution separation of charged analytes from small injection volumes. Coupled to mass spectrometry (MS), it represents a powerful analytical technique providing (exact) mass information and enables molecular characterization based on fragmentation. Although exquisitely sensitive and able to readily capture metabolite classes that the other platforms do not, including nucleotides and highly charged metabolites [101], CE is time consuming and is further hampered by the fact that it covers a range of metabolites that have highly diverse extraction requirements. Therefore, the use of CE-MS in plant metabolism remains relatively rare [74,102]. In addition, CE has poor migration time reproducibility and a lack of reference libraries, which may only be partially overcome by the prediction of migration time [103,104]. However, CE has some distinct advantages over other instruments employed for metabolomics: mainly the facts that it utilizes low separation volume, which is particularly suitable for the study of biological fluids in small experimental animals, and operates under a homogeneous separation environment [105].

### 3.4. Nuclear Magnetic Resonance (NMR)

NMR spectroscopy is a powerful analytical tool that has traditionally occupied a vital position for structure elucidation of natural products [106,107,108,109,110]. Unlike MS, NMR is not discriminatory and is less biased as the results does not rely on the type of ionization condition or the preferences of the used instruments [111]. Hence, this technique allows for the simultaneous detection of the abundant primary metabolites (organic acids, amino acids, and sugars) alongside secondary metabolites (alkaloids, terpenoids, and flavonoids) as typically found in plant natural extracts [112]. In addition, NMR is a very useful technique for the structure elucidation of novel and/or unexpected compounds including those with identical masses and/or different isotopomer distributions [12,113]. Moreover, NMR-based methods are highly reproducible, noninvasive, nondestructive, and require minimal sample preparation as the sample does not get in physical contact with the device as in MS [114]. Another major strength in NMR spectroscopy lies in the direct proportionality between the NMR spectrum signals and the corresponding real molar levels of the detected metabolites, making absolute quantification of all detected metabolites possible without the need for calibration curves of individual analytes, posing it as a powerful tool in quality control purposes of drug extracts [115]. However, a five times delay of the spin–lattice relaxation times (T1) of the slowest relaxing nuclei in the extract is essential in such quantitative experiments to avoid baseline distortions and increase the accuracy of signal integration. This will significantly increase the duration of these experiments compared to other routine NMR metabolomics methods. 

Nevertheless, NMR also has a number of disadvantages in metabolomics analysis [116]. The technique’s major disadvantage is the low sensitivity as the polarization rate of the active NMR nuclei obeys the Boltzmann distribution law. However, recent developments in NMR hardware have at least partially improved this drawback to some extent. The use of superconducting magnets that are commonly operated in metabolomics experiments at field resonance of 600 MHz can increase the sensitivity and resolution of the detected signals [117]. Moreover, the development of cryogenic probes in which the electronics are cooled down to a very low temperature (e.g., 20 K) has resulted in a better sensitivity than standard probes via keeping the noise generated due to random thermal motion of electrons at a minimum [118]. There are currently two types of cryoprobe available, the first is cooled by a closed cycle helium cooler system, which yields a signal-to-noise enhancement up to a factor of five. The second type is a liquid nitrogen-cooled system, commonly used in metabolomics experiments, that is able to provide a sensitivity enhancement of up to a factor of three, but with the capability to be cooled down and warmed up relatively quickly [119]. However, such probes may be of limited relevance for samples with high ionic and/or dielectric conductivity such as those with salts or polar solvents, commonly encountered in plant-derived samples [120]. That said, the low concentrations of natural products remains a limitation of NMR analysis. To partially compensate for this, special probes known as microprobes have been also developed in which the analysis of a few microliters of samples could be possible, thus overcoming the limited availability of the samples [121] as is typical in natural product studies. Dynamic nuclear polarization (DNP) is another interesting advancement that has also been employed to enhance the sensitivity of NMR signals. The fundamental principle behind DNP involves transferring the polarization of the electron spins to the nuclei of interest via microwave irradiation close to the electron Larmor frequency, resulting in a temporary hyperpolarization in this spin-active nuclei (usually naturally abundant ^13^C, ^15^N, and ^1^H). After the polarization transfer step is completed, the sample is transferred to the NMR spectrometer to collect the enhanced (>1000-fold) NMR signals [122].

#### 3.4.1. 2D NMR Based Metabolomics Strategies

Although NMR methods have largely been employed primarily to identify compounds from pure samples as in classical natural product research, they have recently played a larger role in the analysis of mixtures of unfractionated natural extracts [123]. The bottleneck in such complicated analysis is the congregation of the ^1^H NMR spectra, which could be untangled using two main strategies. The first strategy aims to simplify the abundant ^1^H NMR spectra through the use of diffusion and/or relaxation filters or through creating a projection of the ^1^H decoupled spectrum like in 2D ^1^H J-resolved NMR experiments. Relaxation filters are pulse sequences that selectively attenuate, or even remove, signals of components with shorter T1 or spin–spin relaxation times, T2 [124]. Hence, they could be utilized to simplify the ^1^H NMR spectra through selective elimination of signals belonging to high molecular weight or rigid structure (quickly relaxing) compounds. They have also been implemented to attenuate water signals using the water attenuation T2 relaxation Carr-Purcell-Meiboom-Gill (WATR-CPMG) pulse sequence [125]. On the other hand, diffusion filter based experiments such as diffusion ordered spectroscopy (DOSY) commonly simplify ^1^H NMR spectra based on the difference of the translational diffusion coefficients between molecules of different molecular size, achieving what has be described as “NMR chromatography” [126]. The DOSY experiments can be performed in 1D mode, where two or more ^1^H NMR experiments are acquired for compounds possessing different diffusion coefficients or in 2D mode (2D DOSY), where the proton spectrum is depicted in one dimension and the diffusion coefficient is spread in an orthogonal dimension [127]. Interestingly, the extension of 2D DOSY to 3D DOSY is gaining much popularity in metabolomics analysis via incorporating another proton dimension as in 3D COSY-DOSY and 3D TOCSY-DOSY, or another carbon dimension as in 3D DOSY-HSQC [128]. Such DOSY methods have been recently applied in quality control and metabolic fingerprinting of commercial natural extracts [128,129]. Furthermore, they have also been used to identify novel metabolites from natural resources, for example, novel bromopyrrole alkaloids, and agesamides A and B were identified in the crude bromopyrrole fractions from Okinawan marine sponges using the 2D DOSY method [130]. 2D ^1^H J-resolved NMR experiments represent another approach that simplify ^1^H NMR spectra where the overlapping resonance of the ^1^H dimension is resolved through representing the coupling constant of each signal (J value) in a second dimension [131]. 2D ^1^H J-resolved NMR has been widely used in metabolomics classifications studies [24,132], however, prior knowledge of the chemical composition of the studied samples is required, which make this experiment of limited utility in the analysis of novel compound discovery [108]. On the other hand, a newer approach called pure-shift ^1^H NMR spectroscopy has emerged in which better removal of homonuclear J couplings has been achieved [133]. In this experiment, J coupling multiplicities are collapsed into single lines via implementing specific pulse sequences that result in a broadband homo-decoupled spectrum. This method has been applied recently to investigate the metabolome of *Physalis peruviana* fruit aqueous extracts [134]. The simplified data allowed for the identification of glutamic acid, a metabolite not observed in previous studies of the same extract due to the heavy overlap of its NMR signals.

The second strategy that can reliably handle ^1^H NMR overlapping peaks is the spreading of the crowded ^1^H NMR peaks in a second frequency dimension via magnetization transfer [135]. This transfer could be between nuclei of the same type as in H–H correlated spectroscopy (COSY) and H–H total correlated spectroscopy (TOCSY), nuclei of different types such as heteronuclear single quantum coherence spectroscopy (HSQC) and heteronuclear multiple bond correlation (HMBC) experiments, or through space as in nuclear overhauser effect spectroscopy (NOESY) and rotating frame overhauser enhancement spectroscopy (ROESY) experiments [135]. Full details of these common 2D NMR methods that have been used extensively in metabolomics analysis of natural products, and are thus beyond the scope of this review, but can be found elsewhere [12,108,135,136]. It is noteworthy to mention that spreading the crowded signals in indirect proton dimensions such as in COSY and TOCSY results in a relatively short acquisition time due to the high natural abundance of protons, however, some overlap might still exist as a region of only 10 ppm is employed. In contrast, using carbon as a second dimension such as in HSQC and HMBC will require a much longer acquisition time, but allow the resolution of the overlapping signals and the indirect dimension to be increased to more than 200 ppm units. Nevertheless, it should be noted that acquiring data using HSQC or HMBC can aid in the detection of less abundant compounds compared to the direct acquisition from the carbon channel as it is typical in ^13^C-NMR. It is worth mentioning that different pulse sequences have been developed for quantitative HSQC experiments. For example, a new phase modulated pulse sequence called the quantitative, offset-compensated, CPMG-adjusted HSQC (Q-OCCAHSQC) experiment has been proposed to enable the use of HSQC spectra for the quantitative determination of many primary metabolites [137]. However, a combination of different 2D NMR experiments is favored for better analytical performance and enhanced identification strategies (e.g., COSY, (1H,13C)-HMQC, (1H,13C)-HMBC, and NOESY) were used to analyze the unfractionated defensive secretion of the walking stick *Parectatosoma mocquerysi* identifying the novel monoterpene parectadial [138]. COSY, HMQC, and HMBC have also aided in the identification of six terpenoids/sterol secondary metabolites in the crude extract of *Sarcophyton* marine coral [139]. Similarly, a combination of ^1^H, ^1^H COSY, HSQC, and HMBC have identified and quantified structurally related α- and β-bitter acids and their degradation products in hop resin [140] using run times comparable to those used in HPLC. Interestingly, in the same study, HMBC spectra coupled with multivariate techniques were utilized as a novel approach for the classification of 13 commercial hop cultivars. Application of 2D-NMR metabolomics for drug discovery is considerably easier in cases where the secondary metabolites are naturally abundant as typical in the case of hop resin, which contains much lower levels of primary metabolites [54]. In an interesting application of 2D NMR methods, differential analyses of NMR spectra (DANS) has been oriented for natural products drug discovery and comparative metabolomics. DANS uses a simple algorithm that graphically compares the 2D NMR spectra of different biological states to spot the peaks that are discriminatory among this set of spectra. For example, 2D double quantum filtered (DQF)-COSY was employed for DANS analysis of seven different culturing protocols of the filamentous fungus, *Tolypocladium cylindrosporum*, leading to the detection and identification of two novel indole alkaloids, TC-705A and TC-705B, in the unfractionated extracts [141]. Furthermore, DQF-COSY experiments have been applied for the identification of signaling molecules in the model organism *Caenorhabditis elegans* [142] and polyene antibiotic “bacillaene” from *Bacillus subtilis* [143]. On the other hand, multidimensional NMR experiments have already been used to increase the resolution of the newly developed benchtop NMR instruments [144]. Benchtop NMR provides inexpensive and direct NMR access within any metabolomics laboratory, however, they suffer from decreased chemical shift dispersion and peaks overlapping as they are typically operated in the range of 40–80 MHz proton resonance frequency [145]. Benchtop NMR maintain the same J-coupling frequency as the high field instruments since it is independent of the Larmor frequency, making it suitable for some metabolomics and quality control applications, particularly in 2D modes [146]. 

#### 3.4.2. Solid State NMR Based Metabolomics

Another exciting application of NMR spectroscopy is the analysis of semi-solid samples such as fresh plant leaf or intact tissues via the implementation of high resolution solid state magic angle spinning (HR-MAS) NMR [147]. In this technique, the sample is mixed with a minimal volume of solvent. Next, an NMR spectrum is acquired using a special HR-MAS probe rotated at the angle of 54.74° (the ‘magic angle’) and a spinning rate of 4 kHz. HR-MAS NMR provides spectra with a similar resolution to that of classical liquid-state NMR techniques due to the elimination of chemical anisotropies and dipolar coupling that hamper analysis of the solid states samples. This technique requires few sample preparation steps, thus it is gaining much attention in the quality control of herbal medicine and food analysis [148,149]. HR-MAS NMR has been also reported for the metabolic profiling of red algae [150], carrot [151], and Arabidopsis [152] tissues. However, the method is not fully quantitative as the local microenvironment in tissues or cells (e.g., pH) could affect the metabolite chemical shifts, thus hindering its potential, as does the difficulty in incorporating a reference compound [153].

#### 3.4.3. Hyphenated NMR-Based Metabolomics

Whereas standalone NMR spectroscopy has unquestionable merits, on-line hyphenation of separation techniques and NMR spectroscopy (HPLC-NMR) can provide another powerful workflow for de novo identification of novo natural products in crude extracts [154,155]. In hyphenated HPLC-NMR, a flow cell matching the HPLC module is used instead of conventional NMR probes [156]. The separated analytes are eluted from the HPLC column to the crossed flow cell, and their on-line—^1^H NMR spectra are recorded. Some drawbacks were observed in this setup, particularly in the case of applying a gradient mobile phase [157]. Hence, most instrumental setups have changed from on-flow cell to stopped flow HPLC-NMR, and further to the loop/cartridge storage devices that showed wider application in natural product chemistry [158,159] and secondary metabolite identification [160]. Another major development in hyphenated NMR techniques is high-performance liquid chromatography-solid phase extraction-NMR spectroscopy (HPLC-SPE-NMR)where a post-column SPE cartridge is used as an analyte enrichment device prior to NMR acquisition [161]. In this setup, the HPLC eluted component is allowed to be captured onto a solid adsorbent, allowing the HPLC mobile phase to be removed. The adsorbed analyte is then transferred to the NMR probe using the minimal volume of solvents with sufficient elution power (e.g., CD_3_OD, CD_3_CN). The decrease in analyte chromatographic peak volume via the use of minimal solvent confers increased sensitivity of this technique over conventional HPLC-NMR, in addition to significant cost reduction since employing a deuterated HPLC mobile phase is no longer mandatory [162]. However, careful optimization of SPE trapping is necessary from an analytical point of view, as some metabolites may not be trapped onto the SPE stationary phase. In addition, the use of a small volume of deuterated NMR solvents for flushing the required analyte from the SPE stationary phase is a critical parameter that needs to be carefully optimized. HPLC-SPE-NMR has been applied in the structural characterization of secondary metabolites belonging to different classes including flavonoids [163], terpenoids [164], steroids [165], aromatic alkaloids [166], diarylheptanoids [167], iridoids [168], and saponins [169] and has great potential in fields such as chemical ecology in which targeted metabolites are often signaling molecules present at low levels [170]. Furthermore, multiple complementary hyphenated approaches including PDA and MS have been integrated with HPLC-SPE-NMR as versatile platforms for authentication and the structural identification of secondary metabolites directly from extracts [171,172]. The sample is split after the initial HPLC separation with a small portion being routed for PDA and MS analysis and the remainder to the SPE cartridges for collection [173]. HPLC–PDA–MS–SPE–NMR has been used for the dereplication of six novel quinolinone alkaloids, named haplacutine A–F from the *Haplophyllum acutifolium* crude extract [174] and 23 coumarins including six new compounds from the crude ethyl acetate extract of *Coleonema album* leaves [175] and even minor metabolites could also be detected and fully characterized using this advanced platform [176]. These developments thus suggest that NMR, despite its lower sensitivity will retain a role in role in drug discovery in the decades to come.

## 4. Metabolomic Data Processing and Interpretation

GC-MS, LC-MS, and NMR are arguably the most relevant techniques within the context of natural product discovery. GC-MS is a robust platform with great peak resolution in the chromatographic dimension, capable of providing both stable separation and mass spectra fragmentation via its most common ionization source, namely electron ionization (EI). This renders data processing as well as metabolite annotation relatively facile for GC-MS. Well established methods for obtaining robust retention indexes based on series of standards [49], together with the great stability of EI spectra, result in an outstanding machine independent reproducibility of the results obtained and facilitate the use of standard databases for the identification of analytes such as the Golm Metabolome Database (GMD) and MassBank of North America (MoNA), among others [27]. The high resolution of the chromatographic separation and stability of ionization also facilitates the deconvolution of mass features into compound mass spectra and several relatively user-friendly tools are available that are capable of performing all steps from the raw data to peak area and assessment and metabolite annotation matching against databases [177,178,179]. However, the portion of the metabolome that can be covered by GC is significantly limited by the necessary properties of its analytes, namely, being volatile and stable at the high temperatures of analysis. Several compounds can be derivatized to fit such properties, this is unfortunately not the case for most natural products. Moreover, compound annotation relies almost exclusively in matching against known compounds in databases. De novo annotation of compounds based solely on an analyte mass spectra and chromatographic behavior is uncommon. This is because the interpretation of EI spectra is very complex due to the high degree of fragmentation, which can be partially mitigated by the adoption of a milder ionization source such as chemical ionization. Finally, GC has a disadvantage in relation to LC as it cannot be easily translated into a purification technique for further characterization of unknowns by more powerful structural techniques such as NMR. Still, a few interesting attempts have been made in expanding GC-MS structural elucidation such as the work by Matsuo et al. [180]. Here, the authors achieved considerable improvements over traditional spectra and retention index matching approaches by integrating multiple cheminformatics procedures including the use of quality controls to reduce mass spectra background noise and remove artifact peaks, principal component analysis for the selection of biologically relevant variables, EI-MS spectral search in databases, and both retention index filtering and predictions [180]. A further peculiarity of GC-MS is the lack of linearity with respect to quantitation across peaks, requiring quantitative analysis to be made on an analyte by analyte basis [181,182].

LC-MS is the most versatile technique in terms of metabolome coverage and certainly the most relevant for rapid dereplication of natural products in complex mixtures. Its high sensitivity combined with the multitude of stationary phases with different chemistries and ionization sources covering a broad spectra of different compounds allows it to be optimized for nearly every class of natural product. The greatest challenges faced by LC-MS are related to the susceptibility of the results to the many factors influencing separation and ionization efficiency such as changes in solvent composition, column stability, electrospray formation, ion suppression, and other so-called matrix effects. These factors negatively affect spectra and retention reproducibility. The resulting shifts of compound retention times across multiple runs are hard to predict or account for with procedures such as the calculation of retention indexes for GC. The lack of reproducibility in both retention and spectra make the identification of known compounds based on database matches significantly less efficient than for GC. Data processing is also a more challenging task since the lack of ionization stability combined with the lower resolution of LC separation complicates combining mass features into compound spectra, resulting in much more complex datasets with an overwhelming amount of mass features. Despite all of these challenges, the great flexibility of LC-MS can lead to intensive developments for this technique within the context of metabolomics and several pipelines are available to process, analyze, and interpret LC-MS based metabolomics data [32].

Considering that the focus of this review is on the discovery of plant natural products, we mainly describe the processing of untargeted metabolomics experiments, defined as the unbiased processing of all signals within a dataset. Several excellent freeware are currently available for the processing of mass chromatograms with some popular options including XCMS [183], MZmine 2 [184], OpenMS [185], and MS-DIAL [179], all of which have been extensively used for diverse sets of metabolomics data. All of these software work on similar principles, identifying *m/z* signals above a certain threshold as well as the boundaries confining the chromatographic peak representing these signals over time (often referred as features) and returning the respective peak areas/heights. After peak detection, they are usually aligned across multiple samples prior to data analysis. One of the first challenges in processing the resulting data matrixes is the sheer number of features resulting from this process, usually in the order of tens of thousands, much of which are uninformative or redundant, being attributed, for example, to background noise, contaminations and in source fragmentation. Several practices are commonly included in experimental design to assist in the elimination of these signals in the initial steps of data analysis. The inclusion of extraction blanks and technical replicates of quality controls are good examples of such practices and have been shown to be useful for the identification of features that do not originate from the samples or that exhibit low reproducibility [186]. This data cleaning process can significantly facilitate further steps of data analysis, particularly for the identification of features that are most relevant for the separation of experimental groups, for instance, in activity guided fractionation of complex extracts [187]. Another challenging and essential aspect of processing untargeted metabolomics experiments is the identification of features representing fragments, adducts, and isotopes of a same compound. As mentioned before, this process is much more challenging for LC-MS in relation to GC-MS, however, there are a few tools such as CAMERA [188], which can assign putative identity for features based on their retention time and correlation in relation to one another as well as mass differences matching common adducts and in source fragmentation patterns in electrospray ionization.

Particularly relevant for the identification of compounds by mass spectrometry are the experiments including tandem MS fragmentation of either specific ions in a data-dependent acquisition mode (DDA), or of all ions in a data-independent acquisition mode (DIA). Both types of experiment provide second order MS spectra (MS/MS), which are more informative and suitable for compound identification based on matching spectra signals against databases. In DDA experiments, specific ions are isolated and fragmented, therefore all signals in the MS/MS spectra correspond to fragments of the isolated ions. In DIA experiments, larger isolation windows are combined to fragment all ions in first order spectra, therefore there is no link between parental and daughter ions but with the advantage of a comprehensive fragmentation. Several specialized tools as well as extensions to the tools previously mentioned were developed to process such MS/MS data, a few examples including MetDIA [189], which is based on algorithms from XCMS, and MS-DIAL [179], which was developed with a particular focus on processing DIA data despite also being able to process DDA and GC-MS data.

MS/MS spectra are currently the starting point for most mass spectrometry based structural elucidation approaches. The initial procedure usually consists of using the experimental data to search matches through different databases. Several relevant databases for natural product discovery were recently reviewed by Wolfender et al. [190]. We highlight here some of the open source alternatives that are particularly interesting for including curated experimental MS/MS data. Some extensive metabolome databases such as Metlin [191], MassBank [192], MoNA, European MassBank, Global Natural Products Social Molecular Networking resource (GNPS) [28], and Human Metabolome Database (HMDB) [193] include experimental MS/MS data from a large number of natural products from different sources. Additionally, other databases oriented toward specific organisms such as ECMDB for *E. coli*, YMDB for yeast, and ResPect for plants, also provide access to extensive collections of experimental MS/MS data [194]. Most of these alternatives have their own inner structures, hence the functionalities for querying and extracting data vary. An interesting feature that is worth noting for large scale metabolomics is a database integration with other data processing and analysis pipelines. Metlin provides the largest individual collection of MS/MS experimental data acquired in multiple collision energies and despite being the only one of the larger databases mentioned to impose restrictions on downloading data, it is directly integrated with the XCMS online processing platform [191]. MZmine 2 incorporates functions to directly query Kyoto Encyclopedia of Genes and Genomes (KEGG), HMDB, Yeast Metabolome Database (YMDB), LipidMaps, the European MassBank, Chemspider, and Metacyc [184]. MS-DIAL allows for the uploading and automatic matching of spectra against user-defined libraries in the msp. format, the commonly used text-based format for metabolomics spectra [179]. It also provides multiple libraries for download in this format including MassBank, ReSpect, and GNPS, among others.

One of the major obstacles for mass spectrometry based annotation in metabolomics is the lack of characterized standards for the majority of the different compounds found in nature. Therefore, several alternatives have been developed to cope with this limitation. Most large metabolomics databases such as Metlin and HMDB have significantly expanded their coverage by including in silico generated MS/MS spectra of known compounds for which no experimental data are available, usually based on machine learning or quantum mechanics calculations [195]. Similar approaches have also been used to predict putative compounds based on the MS/MS spectra. These functionalities are provided in tools such as CSI:fingerID [196] and MS-FINDER [197]. Another interesting and popular approach provided via the GNPS database is to construct networks based on spectra similarity and extend annotations based on the assumption that related metabolites exhibit similar second order spectra [28].

Additionally to the mass spectra, LC-MS provides another measure with intrinsic structural information, which is the retention time. However, as previously mentioned, there are multiple factors affecting retention time in liquid chromatography that are hard to predict and control to a similar extent of what is done in gas chromatography. Therefore, few approaches for metabolite annotation and cataloguing include retention time information. It is worth mentioning a few recent efforts in integrating this extra information in spectral libraries such as the WEIZMASS [97] as well as strategies for in silico prediction of retention indexes as that provided by PredRet [198].

Finally, there are also approaches for compound annotation that are independent of the acquisition of tandem mass spectra fragmentation. Most untargeted metabolomics platforms nowadays rely on high resolution and high accuracy *m*/*z* measurements provided mainly by QTOF and Orbitrap-based systems. Directly matching putative molecular ion masses against databases is not as reliable as MS/MS spectral matching, it can however, still bring valuable insights in putative structures and the higher accuracy measurements provided by those systems can provide a significantly smaller search space. This is particularly useful when genomics and taxonomic data can be integrated into the search to even further restrict the space of reasonable matches. Unfortunately, this information is still often scarce and not well structured, with few databases providing extensive collections of curated data. One exception worth mentioning is the KNApSack database, which provides the distribution of over 50,000 compounds across over 20,000 different species [199].

## 5. Successful Applications of Metabolomics in Natural Products Discovery

### 5.1. Metabolomics for Secondary Metabolites Identification

In the science of natural product discovery using metabolomics, the separation of metabolites is usually performed using GC or HPLC, which is generally coupled with on-line MS detection. MS fragmentation spectra provide precise information on the structures, and it is thus common to record multiple-stage MS data with the spectra of two or more products (MS2, MSn, where n is the number of production stages) [200]. As above-mentioned, several MS-based databases and software tools are now applied for natural products identification; these databases include the Golm Metabolome Database (GMD) that uses GC retention indices and electron impact (EI) mass spectra, all acquired under defined conditions as mass spectral tags (MSTs) for both neat authentic standards as well as plant extracts in GC-MS data [78]. When GC-MS data of a plant extract are obtained, the MST of its components can be matched to the GMD to record either known metabolites or unique identifiers for unidentified peaks in complex plant extracts. There is also the decision tree search, which predicts substructures of unknown metabolites, even if the full structure cannot be predicted [78]. METLIN, a MS/MS database, includes about 62,000 spectra representing more than 12,000 metabolites. These spectra were acquired under standardized conditions using electrospray ionization in positive and negative ionization modes on a quadrupole time-of-flight (QTOF) mass spectrometer [201]. The limitations of this database are the lack of chromatographic data and closed design. Meanwhile, MassBank is a database of high resolution MS-based platforms (GC-MS and HPLC-MS). All MassBank has spectral information, and some have chromatographic information, but there are no retention times (or indices) data [192]. In contrast, ReSpect is an MS2 database specific to plant metabolites. The main advantage of this database is that spectral records are annotated with taxonomic information about the species from which a particular metabolite has been extracted and to which structural class the metabolite belongs [202]. Similarly, the Global Natural Products Social Molecular Networking resource (GNPS) is an MS2 database for natural products [28]. Several databases are considered as an important tool to facilitate the dereplication process [80,203]. 

NMR is the most powerful technique with respect to structure elucidation, as it is highly reproducible, quantitative, requires simple sample preparation, and is able to measure analytes over a wide range of solvent conditions [204]. It is, however, limited by being slower and less sensitive than MS, and as such, it cannot easily be used as a routine technology in metabolomics. Instead, its application is mainly limited to measuring the most abundant metabolites in the sample [205,206]. In natural product discovery, fractions with the metabolites of interest are separated through chromatographic runs and then subjected to off-line NMR analysis. It is more common in applications of metabolomics to make NMR analysis of plant extracts or biological fluids without chromatographic separation [80]. The applications and limitations of NMR databases that are particularly useful for natural products discovery are discussed in Table 1. To search in one of the mentioned NMR databases, the obtained spectra should be processed and interpreted using external software and entered into a web interface as a peak table. The rNMR software interfaces convert spectral peaks to a searchable file format, so can be used in the MMCD database [207]. On the other hand, SpinAssign is used for the identification of compounds using the 13C-HSQC spectra of complex cell extracts [208], while the MetaboHunter and COLMAR tools are designed to search the NMR spectra of mixtures against those of a subset of metabolites in HMDB and BMRB [209,210]. 

There is an increasing tendency in metabolomics to analyze the same sample by both NMR and MS. Taking advantage of both methods enables a better coverage of the metabolome [217,218]. NMR allows the identification of trends in metabolic alteration across core metabolic pathways, while MS provides the identification of the minor metabolites. The integration of NMR and MS can increase the size of the obtained dataset with the added complexity of the simultaneous processing, analysis, and interpretation of two dissimilar data types [218,219]. Indeed, as described below, LC-online-NMR has been proposed as a route toward improving coverage of the metabolome. However, despite the advocacy of this approach, relatively few examples of its utility have been published to date [106,114].

### 5.2. Metabolomics in Defining or Refining the Pathway Structure of Plant Natural Products

Given that this subject has been extensively reviewed [3], we will keep this section short, suffice to say that the chemistry of many medicinal plants has remained largely unexplored. Despite the advances of synthetic biology for the production of medicinal compounds in heterologous hosts (described in the section below), the native plant species often remain the most reliable and economic source for their production. It is thus of fundamental importance to investigate the metabolic composition of medicinal plants to characterize their natural metabolic diversity and to define their in planta biosynthetic routes in order to develop strategies to further increase their content. In this vein, the cases of benzoisoquinoline and monoterpenoid indole alkaloids, cannabinoids, caffeine, ginsenosides, withanolides, artemisinin, and taxol represent interesting case studies in which the contribution of recent integrative metabolomics and genomic approaches in augmenting earlier biochemical strategies have helped to elucidate their metabolic pathways and cellular compartmentation [3].

### 5.3. Metabolomics to Aid Metabolic Engineering of Secondary Metabolites Production

A recent approach in the field of metabolomics is its use as a generic debugging tool for engineered microbial production systems that arise as a result of advanced synthetic biology. The synthetic biology of secondary metabolite production is not restricted to the awakening of the cryptic metabolite production encoded in newly sequenced genomes, but it also serves in modifying and recombining the modular biosynthesis apparatus found in nature, which leads to the generation of novel chemistry, in addition to the more ambitious re-engineering strategies such as refactoring, which means replacing the native regulatory machinery by fine-tuned designer systems or the improvement of production levels through the redeployment of primary metabolism [220]. 

In a metabolomics study of the consequences of an overexpression of ncRNA-based regulatory element in *Streptomyces coelicolor* [221], where ncRNA affected not only the nearby metabolites, but also the metabolite levels that showed rapid changes throughout the metabolic network of the organism. The reproducibility of the metabolite dynamics was ensured by repeated metabolite profiling. Another application is the detection of the accumulated toxic side products and intermediates, or the depletion of required precursors, which is considered as metabolic bottlenecks. In these cases, metabolomics provides an unbiased overview of the metabolic status of a system and its changes due to the overproduction of compounds of interest, which in combination with systems biological modeling can drive cycles of refined engineering [222]. The *Streptomyces lydicus* metabolome was characterized in various cultures where the critical precursors of streptolydigin were identified through the depletion in overproducing cultures. Here, the added precursors (glutamic acid and proline) led to an increase in streptolydigin production, with no effect on the strain growth. Therefore, metabolomics approaches enabled the identification of the major bottleneck in the system with no need for understanding the underlying metabolic and regulatory network [223].

Given the massive diversity of secondary metabolism in plants, a recent approach to metabolic engineering has been combinatorial biosynthesis, an engineering strategy that allows for the generation of novel, structurally similar, but distinct compounds, which is achieved by combining biosynthetic genes of related metabolic pathways from different organisms in a single host [224,225,226]. This requires the use of enzymes that have relaxed specificity and thus are able to incorporate derivatives of their natural substrates. Other than pioneering studies on the synthesis of novel carotenoids in nonphotosynthetic *Escherichia coli* [227], a few other reports of successful targeted combinatorial biosynthesis of (novel) terpenoids in plants exist. For example, the biosynthesis of monoterpenoid indole alkaloids (MIAs) in the Madagascar periwinkle (*Catharanthus roseus*) was carried out following the expression of two different bacterial halogenases, yielding new-to-nature halogenated MIA derivatives [228]. Similarly, biosynthetic genes of five different plants were combined in yeast to produce a monoglycosylated triterpene saponin that does not occur in any of the parent plant species [229]. Moreover, artemisinin was recently transferred from the medicinal plant of origin into the crop plant tobacco [230]. In all of the examples above, metabolomics was instrumental in confirming the novel natural products. It is important to note that this subject area is currently in bloom and it seems reasonable to anticipate that many more examples will follow shortly. Furthermore, the labelling approach in metabolomics such as ^13^C-based metabolomics studies, can also be applied for discovering novel secondary metabolism [231,232,233]. Although this technique needs a single source of carbon or nitrogen, this can be achieved by tracing the distribution of a class-specific precursor to side branches of the metabolic network [234]. 

### 5.4. Metabolomics and Dereplication

Another application of metabolomics is the dereplication of the natural product biosynthesis at different development stages through the use of various analytical methods, the bioassay guided isolation, thus rapid dereplication of known activities is efficiently delivered [235]. High resolution Fourier transform mass spectrometry (HRFTMS) is usually used for dereplication of secondary metabolites together with LTQ-Orbitrap and high resolution NMR. Identification of high resolution MS and NMR of the metabolomes is undertaken using the online and in-house databases. Multivariate analysis allows the Fourier transformation of the FID (free induction decay) of multiple samples data, in order to statistically validate the parameters to produce biologically novel secondary metabolites [236,237]. Combining PCA with dereplication and molecular networking was applied on microbial extracts for the discovery of novel compounds where the molecular networking provided a chance to compare the metabolic profiles of complex crude fermentation extracts, leading to efficient chemical dereplication [238].

The dereplication process was summarized by Tawfike et al. (2013, Figure 2) where the extracts, fractions, and purified secondary metabolites were first tested for possible biological activity. After that, the chemical profiling of the active extract or fraction was analyzed using high resolution mass spectrometry and NMR spectroscopy to identify the constituents of interest at an early stage to minimize the efforts by focusing on the active principles. Following the application of various metabolomic tools and multivariate statistics, the obtained huge datasets could be utilized for the identification of the biomarkers of the biologically-active extracts. The identified biomarkers can then be employed in the production of the novel secondary metabolites to provide sustainability of the interesting metabolites in genetic and metabolic engineering [113].

In this section, we will discuss some examples for the application of metabolomics in dereplication. Dereplication was applied for the identification and quantification of tocopherol using HPLC analysis of Brazil oil nut from different geographic locations in Brazil. This study proved the presence of the different amounts of α- and β-tocopherol in some samples with the absence of tocopherols in other samples [239]. Dereplication was also used to select superior banana genotypes for breeding based on the phenolic contents and carotenoid profiles, where the HPLC analysis showed an appreciable amount of pro-vitamin A carotenoids in the active germosplasm samples compared with the main cultivars that are currently marketed [240]. Coffee genotypes of three different regions in Brazil were distinguished using GC-MS coupled with statistical analysis where 44 metabolites were characterized as the chemomarkers for differentiation of the origin and genotype [241]. Design of experiments (DoE) and partial least squares (PLS) were used to prove that the physicochemical properties of the solvent have a significant effect on its extraction capacity [242]. Moreover, dereplication was used to detect bromotyrosine-derived metabolites in 14 species of sponge belonging to genus *Aplysina* through measuring their UV absorption using LC-photodiode array detector (PDA)-MS analysis [243]. Additionally, dereplication could differentiate between green and brown Brazilian propolis bead on their terpenoid content using GC-MS [244] as well as the discovery of biomarkers and novel active constituents that may be active against different neglected diseases [245]. UHPLC-(TOF)-MS, together with hierarchical clustering analyses (HCA), was applied to differentiate between six *Lippia* species based on their chemical constituents [246]. In addition, dereplication was successfully applied to determine 11 anti-inflammatory biomarkers in 57 extracts of Asteraceae leaves using HPLC-MS among 1241 peaks [247]. Similarly, 44 metabolites were identified as chemomarkers to determine the geographical origin and genotype of coffee in Brazil using GC-Q-MS [241]. 

### 5.5. Metabolomics for Quality Control of Natural Products

Metabolomics is also finding utility in the quality control of natural products, being used to monitor the variation of metabolic profiles among individuals, environmental alterations during growth and harvesting, post harvesting treatment, extraction, and method of isolation. The change in the chemical profiles have a significant effect on the efficacy of phytomedicines prepared from these herbs [18]. Unsupervised principal component analysis (PCA) and supervised partial least square analysis/partial least square analysis with discriminant analysis (PLS/PLS-DA), combined with ^1^H-NMR, are the most common techniques used in quality control. These methods were used to study the effect of location on the percentage of various constituents of chamomile (*Matricaria recutita* L.) [248]. Determination of ∆^9^-tetrahydrocannabinolic acid (THCA) and cannabidiolic acid (CBDA) was carried out to discriminate between different *Cannabis sativa* [249]. Similarly, discrimination of different samples of St John’s Wort (*Hypericum perforatum*) from different batches of the same supplier, according to the variation of the content of the antidepressant flavonoids, has also been reported [250]. Metabolomics has also been applied to detect adulterations of herbal preparations with similar species but with low levels of constituents responsible for the activity. A company produced antimalarial capsules containing *Artemisia afra*, which is free from artemisinin instead of *Artemisia annua*, and this could be easily detected by ^1^H-NMR and PCA [251]. Metabolomics can additionally be used to profile the biofluids of individuals who have been medicated with natural products and a vast literature describes such studies [30,252,253].

## 6. Conclusions and Future Perspectives

Metabolomics experiments offer an improved expedited route for plant natural product drug discovery and is increasingly applied in a bioactivity-guided approach from natural extracts. This review provides an overview for the most widely employed analytical tools in metabolomics experiments for natural products’ drug ability viz. GC-MS, LC-MS, CE-MS, and NMR with recent advances in their applications. Furthermore, different sample preparation protocols, data processing tools, and some successful applications of metabolomics in natural products discovery are outlined, highlighting its advantages and limitations. Although metabolomics has clearly transcended the classical approach of lead compound discovery as previously mentioned, the main challenge in such experiments appears to simultaneously explore the extreme complexity and the huge chemical diversity of metabolites present in any natural organism. In addition, the annotation of metabolites in such complicated environments remains another obstacle that needs to be tackled. The establishment of organism databases and sharing of spectral data in public repositories will aid in that regard, and avoid dereplication of already existing metabolites in bioactivity guided studies for active agents. Bioinformatic tools that can link the metabolomics dataset with bioactivity results have yet to be implemented for all spectral datasets. Finally, we believe that there is urgent need to apply the integrative approaches of genomics and metabolomics to understanding the metabolism of natural products in plants and elucidate their metabolic pathways. 

## Figures and Tables

**Figure 2:**
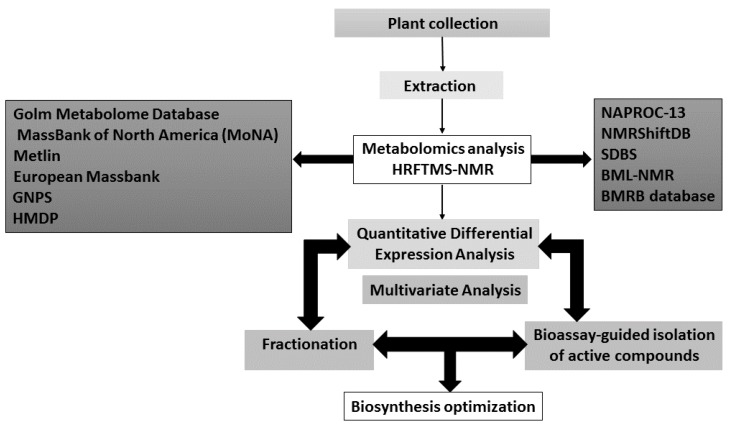
A flowchart showing the application of metabolomics in dereplication.

**Table 1:** Nuclear magnetic resonance (NMR) databases and computational methods.

Database	Applications	Notes	References
NAPROC-13	Is useful in cases of dereplication as it has more than 20.000 of natural product spectra and inclusion of metabolite classification	Its closed design, there is no user access to spectral data. ^13^C-NMR spectra, molecular formula or predicted molecular weight can only be searched separately.	[211]
NMRShiftDB	Not limited to NP and has 51,000 collected spectra. It accepts submissions	It lists NMR chemical shifts, but not peak size.	[212,213]
SDBS	Not limited to NP and has 29,000 collected spectra. It uses multiple kinds of spectra in a single search.	Does not accept submissions.	[214]
BML-NMR	The spectral depth of 16 different one- and two-dimensional experiments for each compound provides high quality references	Covers only 208 compounds, but each compound is measured with 16 different NMR parameter sets which provides high quality references. These metabolites were selected based on their importance within metabolic pathways and their detection potential by NMR. They were analyzed at pH 6.6, 7.0, and 7.4.	[215]
BMRB database	Contains NMR data for various biomolecules with a focus on protein, peptide, and nucleic acid spectra. Spectra are available for downloading in raw and processed data formats	The database mainly covers the compounds involved in the lignin biosynthesis which is a plant cell wall constituent and the compounds obtained by plant cell wall deconstruction.	[216]

NAPROC-13: Carbon-13 Database of Natural Products and Related Substances; DB: Database; SDBS: Spectral Database for Organic Compounds; BML-NMR: Birmingham Metabolite Library; BMRB: Biological Magnetic Resonance Data Bank.
